# A meta-analysis reveals complex regulatory properties at Taf14-repressed genes

**DOI:** 10.1186/s12864-017-3544-6

**Published:** 2017-02-16

**Authors:** Josipa Nemet, Nikolina Vidan, Mary Sopta

**Affiliations:** Department of molecular biology, Ruđer Bošković Institute, Bijenička 54, Zagreb, Croatia

**Keywords:** *Saccharomyces cerevisiae*, Transcription regulation, Meta-analysis

## Abstract

**Background:**

Regulation of gene transcription in response to stress is central to a cell’s ability to cope with environmental challenges. Taf14 is a YEATS domain protein in *S.cerevisiae* that physically associates with several transcriptionally relevant multisubunit complexes including the general transcription factors TFIID and TFIIF and the chromatin-modifying complexes SWI/SNF, INO80, RSC and NuA3. *TAF14* deletion strains are sensitive to a variety of stresses suggesting that it plays a role in the transcriptional stress response.

**Results:**

In this report we survey published genome-wide transcriptome and occupancy data to define regulatory properties associated with Taf14-dependent genes. Our transcriptome analysis reveals that stress related, TATA-containing and SAGA-dependent genes were much more affected by *TAF14* deletion than were TFIID-dependent genes. Comparison of Taf14 and multiple transcription factor occupancy at promoters genome-wide identified a group of proteins whose occupancy correlates with that of Taf14 and whose proximity to Taf14 suggests functional interactions. We show that Taf14-repressed genes tend to be extensively regulated, positively by SAGA complex and the stress dependent activators, Msn2/4 and negatively by a wide number of repressors that act upon promoter chromatin and TBP.

**Conclusions:**

Taken together our analyses suggest a novel role for Taf14 in repression and derepression of stress induced genes, most probably as part of a regulatory network which includes Cyc8-Tup1, Srb10 and histone modifying enzymes.

**Electronic supplementary material:**

The online version of this article (doi:10.1186/s12864-017-3544-6) contains supplementary material, which is available to authorized users.

## Background

Fine-tuning of gene expression has a central role in cellular adaptation allowing cells to thrive under different environmental challenges. A fundamental physiological challenge for the cell is to balance energy efficient growth and rapid response to environmental stress. Regulation of gene transcription is a key control mechanism in the regulation of gene expression. Emerging data reveal two distinct regulatory mechanisms that cells use to moderate transcription of stress-related and growth-related genes [1]. Studies in the yeast *S. cerevisiae* have established a number of principles relevant to these global modes of regulation. Growth-related and housekeeping genes tend to have promoters which lack a TATA box and their transcriptional control is dominated by the TFIID complex. In contrast, stress-related genes generally contain TATA boxes in their promoters and are predominantly regulated by the SAGA complex. Stress-related genes are extensively regulated, prefer negative versus positive regulation by chromatin and possess higher plasticity of expression [2, 3]. A genome-wide study of the structure and organization of eukaryotic pre-initiation complexes (PIC) demonstrated that there are also distinct functions of the +1 nucleosome at these two classes of genes [4]. Nucleosomes and PICs assemble cooperatively at TATA-less/TFIID dependent promoters, in which case the +1 nucleosome is instructive for transcription start site (TSS) selection. In contrast, at TATA-containing/SAGA dependent promoters nucleosomes and PICs assemble competitively and this allows for greater stochasticity or plasticity of expression, wherein nucleosome loss primes the gene for a high level of transcription or vice versa [4].

In yeast, Taf14 is one of three YEATS domain proteins which include Yaf9 and Sas5 [5]. While *TAF14* is a non-essential gene, disruption of *TAF14* causes sensitivity to temperature, osmolarity, and DNA damaging agents such as UV light, gamma-irradiation, MMS, 4NQO and hydroxyurea [6–8]. Taf14 is a subunit of several chromatin remodeling complexes, SWI/SNF, INO80 and RSC, the histone acetyltransferase NuA3 and the general initiation factors, TFIID and TFIIF [9–11]. With the exception of SWI/SNF, Taf14 has been shown to directly interact with the catalytic subunits of the remaining five complexes [9]. This distribution of Taf14 among chromatin-modifying complexes and general transcription factors suggests an important role in the regulation of gene transcription. Indeed, early studies showed that disruptions of the normally non-essential transcription elongation factor *PPR2* (TFIIS) are lethal in conjunction with deletion of *TAF14* [12]. Similarly dosage compensation of a *GAL4* deletion by Gal11 requires an intact Taf14 [13]. In *S. pombe* Taf14 (alias Tfg3) was found to be required for stress responsive transcription [14] while more recently RNA-seq analyses of gene expression in a *S. cerevisiae taf14* mutant identified a number of gene categories that are affected by *taf14* mutation [15].

The YEATS domain is a conserved domain found in over 100 proteins across at least 50 distinct species [5]. The structure of the Taf14 YEATS domain indicates that it adopts a compact and rigid fold which may function as a scaffold in chromatin remodeling and transcription complexes [16]. Studies on the human Af9 YEATS domain and the Taf14 YEATS domain show that it acts as an acetyl-lysine reader domain, showing a high preference for H3K9ac binding [15, 17]. Furthermore, disruption of the Taf14-H3K9ac interaction was shown to impair transcription in vivo [15]. More recently, both Taf14 and AF9 YEATS domains have been shown to recognize crotonylated histone H3 [18, 19] and in the case of AF9 this interaction has been associated with active gene expression [19].

In this report we analysed published genome-wide transcriptome and ChIP data to look at Taf14 dependent effects on transcription and to discern the coregulatory factors that impact gene expression in combination with Taf14. We find that particularly for Taf14-repressed genes multiple levels of regulation are apparent and suggest new interactions between Taf14 and specific factors that negatively regulate gene transcription.

## Methods

### Data preparation

The raw data files from previously reported microarray analysis of wild type and *taf14*Δ strains [20] have been downloaded from the Gene Expression Omnibus Database (www. ncbi. nlm. nih. gov/geo), accession number GSE12150. Raw CEL files were quintile normalized using GCRMA followed by baseline transformation to the median of all samples. The data was then filtered for expression values to exclude the genes that showed less than 20th percentile expression in all samples of at least one of the four conditions. A two-way ANOVA for cell line and treatment was run and genes that passed significance analysis at a p-value <0,05 after a Benjamini Hochberg False discovery rate correction were selected for further analysis. A twofold cut-off filter was then applied to select genes that showed differential expression. In the *taf14*Δ mutant, 205 genes showed increased expression (hereafter referred to as Taf14-repressed genes) and 210 genes showed decreased expression (referred to as Taf14-induced genes) relative to the wild type strain (Additional file 1: Table S1).

We collected 28 gene expression profiles from 14 publications. The profiles were obtained for strains exposed to different non-standard growth conditions or strains mutated for various transcription factors. The complete list of publications and experiments is available in Additional file 1: Table S2. Data were downloaded from papers’ web supplements. In results “positively regulated” is defined as those genes that decrease the most (10th percentile cutoff) when the indicated factor is deleted; “negatively regulated” is defined as those genes that increase the most (top 10th percentile) when the indicated factor is either deleted or mutated as indicated. 10th percentile cutoff was also used for expression data for different stress conditions.

For occupancy and co-occupancy analysis data were obtained from [21]. Occupancy values meeting a 5% FDR cutoff provided by authors were deemed to be significant and for increased and decreased occupancy upon heat-shock, top and bottom 10th percentile cutoff was used for comparisons.

The number of genes in each data set exceeds 2000.

### Data analysis

An unbiased set of genes or promoters is expected to have a distribution as observed throughout the entire genome. Values significantly higher than the genome-wide distribution reflect a bias toward regulation by a distinct factor. With this type of analysis, data that are not relevant will show low significance, meaning that the distribution of an analysed property is very similar to the genome-wide average. *P* values, representing differences between genome-wide distributions and distributions in analysed samples, were calculated using the CHITEST formula in Excel, converted to a log scale and the absolute value taken. Negative signs are inserted where overlap was lower than that expected by chance. For clarity, any absolute value less than 4 was replaced with three periods.

## Results

### Genes negatively regulated by Taf14 tend to be stress related, SAGA-dominated and contain a TATA box

In the early stages of transcription the TATA-binding protein (TBP) must be recruited to core promoters either by TFIID or SAGA complex [3]. A genome wide study revealed that 9–10% of all genes prefer SAGA complex for TBP recruitment to promoters, therefore an unbiased population is expected to have a distribution similar to this genome-wide average [3]. One of the first motifs described in a core promoter is the TATA box, however later genome wide studies showed that the TATA box is present in only 20% of all *S. cerevisiae* genes [2]. In Additional file 2 we list the percentages of representation for all analysed properties in the genome as well as in the specified extracted gene sets. A genome wide study that compared these two traits, presence of TATA box and preferance for either TFIID or SAGA for TBP recruitment, revealed that SAGA usually loads TBP on promotors of TATA-containing genes, whereas TFIID is dominant among TATA-less genes [3]. We found that genes that increased the most in a *taf14* deletion strain were strongly biased toward being SAGA-dominated (Table 1, row 1). Strikingly, compared to 9–10% of SAGA-dominated genes present in an unbiased population, we find that among genes that were significantly induced in a *taf14* deletion strain, 46% of those genes were previously described as SAGA-dominated (Additional file 2: Sheet “Taf14”). In accordance with the SAGA dependence of Taf14 negatively regulated genes, we find that this group of genes also tend to contain a TATA box (Table 1, row 2). Taf14 negatively regulated genes significantly overlap with a group of genes whose transcription is at low level during normal growth conditions (Table 1, rows 5 and 6) but interestingly tend to require rapid and variable regulation under inducing conditions (Table 1, row 7). In addition, we find a significant overlap of Taf14 negatively regulated genes with genes repressed by evolutionary pressure, suggesting that they are prone to fast evolution of gene expression control (Table 1, row 8).

**Table 1:** Significance values relating general genomic properties to Taf14 regulated genes^a^

Row		Negatively regulated by Taf14		Positively regulated by Taf14	
General properties					
1	SAGA dominated	62		…	
2	TATA-containing	46		6	
3	TATA and SAGA-regulated	82		…	
4	TATA-less and TFIID-regulated	−48		−8	
Transcription dynamics		10th UP	10th DOWN	10th UP	10th DOWN
5	Transcriptional rate (standard conditions)	…	10	…	…
6	Expression intensity (standard conditions)	…	10	…	…
7	Expression intensity (inducing conditions)	23	…	…	…
8	Evolutionary pressure	…	17	…	…

^a^Detailed information with calculations and percentages are provided in Additional file 2: Sheet “Taf14”

A number of previous studies identified a bimodal regulation of the transcriptome showing that expression of growth- and stress-related gene expression programmes utilize distinct sets of regulators [1, 3]. In *S. pombe* Taf14 (alias Tfg3) has been shown to be required for stress responsive gene transcription [14]. In addition, *taf14* deletion strains have been shown to be sensitive to a variety of stressors [6–8]. We therefore analyzed previously published large scale environmental stress data [22, 23], and found that genes that are commonly upregulated during stress, including heat, acidity, high osmolarity, DNA damage, oxidation and carbon or nitrogen starvation were significantly represented in the group of genes negatively regulated by Taf14 (Table 2, row 1–8). Among genes positively regulated by Taf14 we find no significant overlap with stress-induced or repressed genes except in the case of MMS dependent DNA damage where we a find strong correlation of genes positively regulated by Taf14 and genes induced during exposure to alkylating agent methyl methanesulfonate (MMS) (Table 2, row 8). The results show that many stress-responsive genes are negatively regulated by Taf14 under normal growth conditions and that at least for the case of MMS Taf14 is required for both repression under normal conditions and induction of MMS responsive genes upon exposure to MMS (Table 2, row 9). As there is no data for other stressors in strains lacking Taf14 it remains to be determined whether this is a general property of Taf14 under all stress conditions.

**Table 2:** Significance values relating genomic properties to Taf14 regulated genes^a^

		Negatively regulated by Taf14		Positively regulated by Taf14	
Row	Condition	10th UP	10th DOWN	10th UP	10th DOWN
1	NaCl	76	…	…	…
2	Sorbitol	62	…	…	…
3	Acid	24	5	…	…
4	H2O2	23	…	…	…
5	Heat	38	…	…	…
6	Diauxic shift	22	…	…	…
7	Amino acid starvation	6	…	…	…
8	MMS	69	…	…	35
9	Δtaf14 (MMS)*	…	127	…	…

^a^Detailed information with calculations and percentages are provided in Additional file 2: Sheet “Taf14”

### Heat shock leads to changes in the composition of the transcription machinery at a significant number of Taf14-repressed promoters

Genome-wide transcriptional studies in yeast reveal that within 15 min after exposure to heat shock, the cellular transcriptome undergoes substantial perturbations, involving transient repression of 600 genes and activation of 300 genes [22]. Venters and coworkers determined the genome-wide binding locations of 200 transcription-related proteins under normal and acute heat-shock conditions [21]. We used these chromatin immunoprecipitation (ChIP) data to analyse which regulators occupy promoters of Taf14-impacted genes under normal conditions and also to determine the most common changes in composition of regulators found at these promoters upon heat shock. We found no significant changes in the composition of regulators after heat shock when we looked at Taf14-induced genes. However, when we looked at Taf14-repressed genes, we found as expected an over-representation of promoters with increased occupancy of Pol II and the general initiation factors, as well as decreased occupancy for the Htz1 histone-repressive mark, all of which indicate increased expression among these genes after heat shock (Table 3, rows 1–9). We also found a significant overlap of Taf14-repressed genes with promoters that showed the largest increase in occupancy of INO80, NC2 and Cyc8/Tup1 (Table 3, rows 10–15).

**Table 3:** Significance values relating whole genome occupancy changes in response to heat shock to set of genes repressed by Taf14 under normal conditions^a^

			Occupancy change upon heat shock	
Row	Protein	Complex	10th UP	10th DOWN
1	Rpb3	Pol II	27	…
2	Rpb7		18	…
3	Rpo21		10	…
4	Sua7	TFIIB	43	…
5	TAF1	TFIID	7	…
6	Bdf1		17	…
7	Tfa1	TFIIE	41	…
8	Tfg1	TFIIF	29	…
9	TBP	TFIID, SAGA	11	…
10	Bur6	NC2	16	…
11	Ncb2		12	…
12	Cyc8	Cyc8-Tup1	12	…
13	Skn7		8	…
14	Ino80	INO80	11	…
15	Taf14		9	…
16	Htz1	nucleosome	…	10

^a^Detailed information with calculations and percentages are provided in Additional file 3: Sheet “Other analyses”

We further analysed promoters that showed the highest change in Taf14 occupancy upon heat shock (Table 4). We found that genes with the highest increase of Taf14 occupancy often contained a TATA box and overlapped with genes induced during different stress conditions. Whereas in the genome the representation of TATA-containing promoters is approximately 20%, among genes with the highest increase in Taf14 occupancy 34% are TATA-containing genes (Additional file 3: Sheet “Other analyses”). Genes with the highest increase of Taf14 occupancy tend to be positively regulated by SAGA complex and the stress-related activators Msn2/4 and negatively by INO80, NC2 and Srb10 kinase and Mediator tail module. Promoters with the largest decrease in Taf14 occupancy (Table 4) after heat shock generally show correlations that correspond to previously described TFIID-dominated genes [3], meaning high transcription rate under standard conditions, dominance of TFIID (Table 4, row 2 and 3) and overrepresentation of genes that are repressed under different stressful conditions (Additional file 3: Sheet “Other analyses”).

**Table 4:** Significance values relating genomic properties to promoters with significant Taf14 occupancy change in response to heat shock^a^

Row		Increased Taf14 binding	Decreased Taf14 binding
General properties			
1	TATA containing	9	…
2	SAGA-dominated	13	−4
3	High transcriptional rate	…	29
4	Short mRNA half life	…	7
5	Repressed by evolutionary pressure	5	…
Increased expression during environmental stress			
6	Heat	11	…
7	H2O2	5	…
8	diauxic shift	10	…
Negatively regulated by			
9	Taf14	9	…
10	Ino80	4	…
11	Bur6	7	…
12	MEDtail	6	…
13	Srb10 kinase	4	…
Positively regulated by			
14	Msn2	7	…
15	Msn4	7	…

^a^Detailed information with calculations and percentages are provided in Additional file 3: Sheet “Other analyses”

In Table 5 we show the correlations of promoters with significant changes in Taf14 occupancy and different transcriptional regulators. When we looked at increased binding of components of the SAGA-regulatory pathway [1] we mainly find correlations with promoters that show increased Taf14 binding (Table 5, rows 2–18). A common regulatory role of Taf14 and factors from the SAGA pathway on promoters of stress-related genes is further confirmed by the high overlap of their mutant transcription profiles (Additional file 1: Table S3). On the other hand, we found a correlation of promoters with decreased binding of Taf14, Pol II, TFIID and other general transcription factors (GTFs) during a shift to 37 °C (Additional file 1: Table S4), due to a general transcription role of Taf14 in transcription of growth related and housekeeping genes most likely through its association with TFIID and TFIIF.

**Table 5:** Significance values relating genomic properties to promoters with significant Taf14 occupancy change in response to heat shock^a^

			Increased Taf14 binding		Decreased Taf14 binding	
Row	Protein	Complex	10th DOWN	10th UP	10th DOWN	10th UP
1	TBP	TFIID, SAGA	…	25	94	…
2	Bur6	NC2	…	24	18	…
3	Ncb2		…	60	42	…
4	Ada2	SAGA	…	36	…	…
5	Gcn5		…	16	…	…
6	Chd1		…	27	…	…
7	Spt20		…	23	…	…
8	Spt3		…	37	…	…
9	Ino80	INO80	…	48	…	…
10	Swi3	SWI-SNF	…	8	…	…
11	Rsc1	RSC	…	19	…	…
12	Rsc8		…	17	…	…
13	Cyc8	Cyc8-TUP1	…	9	6	…
14	Tup1		…	14	…	…
15	Skn7	SKN7	…	33	…	…
16	Med2	Mediator	…	33	…	…
17	Rgr1		…	12	…	…
18	Srb5		…	7	…	…
19	Top1	TOP1	…	10	…	…
20	Htz1	Nucleosome	10	…	…	34

^a^Detailed information with calculations and percentages are provided in Additional file 3: Sheet “37-25UTmax”

Genes that are induced by *TAF14* disruption under normal conditions strongly overlap with genes that show increased Taf14 occupancy upon heat shock (Table 4, row 9). We analysed in detail this subpopulation of Taf14 repressed promoters that also show a significant increase of Taf14 occupancy in response to heat shock and find that most of the high significance values of properties from tables 3 to 5 are even more prominent in this filtered population of genes (Additional file 1: Table S5).

In order to further distinguish between general and specific regulatory roles of Taf14 in genome-wide transcription, we analysed the location of Taf14 before and after heat shock. Table 6 shows that genes with decreased Taf14 binding upon heat shock have Taf14 primarily bound in the transcription start site (TSS) region prior to heat shock where the general transcription machinery usually binds [21]. However, genes with increased Taf14 binding upon heat shock are primarily those that have Taf14 bound to the upstream activating sequence (UAS) region after heat shock (Table 6) where proteins of the SAGA stress-related pathway tend to reside [21]. When we examined Taf14-repressed genes in the same manner we find that the only signficant correlation with Taf14 occupancy is that of UAS binding after heat shock (Table 6).

**Table 6:** Significance values relating whole genome Taf14 binding location to set of promoters with distinct properties^a^

		Taf14 occupancy change in response to heat shock		Taf14-repressed genes with increased Taf14 occupancy after heat shock
		Decreased binding	Increased binding	
Taf14 bound over 5%FDR	25UTmax	35	…	…
	25UAS	7	…	…
	25TSS	53	…	…
	37UTmax	…	36	7
	37UAS	…	35	8
	37TSS	…	10	…

^a^Detailed information with calculations and percentages are provided in Additional file 3: Sheet “Other analyses”

### Taf14 negatively regulated genes are highly regulated by nucleosomal- and TBP-targeted mechanisms

Transcriptional regulators can be classified into three broad groups depending on whether they regulate chromatin, TBP or pol II [24]. We examined transcriptional regulators belonging to each of these three groups to ascertain whether any displayed a preference to positively or negatively co-regulate transcription with Taf14.

We examined the two classes of Taf14-dependent genes, either negatively or positively regulated, in order to identify whether they contain a greater content of genes impacted by a certain regulator than would be expected from the genome-wide distribution. In Additional file 1: Table S1 we list all genes found to be either negatively or positively regulated by Taf14 with related functional categories and gene ontology.

Taf14 has been found to be a subunit of three different ATP-dependent chromatin remodeler complexes, INO80 [25], SWI/SNF and RSC [9, 10] and we first examined the relationship of genes regulated by these remodelers to those regulated by Taf14. As expected, we found a very strong overlap of Taf14-repressed genes with genes repressed by Ino80 (Table 7, row 7). We found a smaller but significant overlap with genes repressed by Swi2 and no significant overlap with Rsc30 repressed genes (Table 7, row 8,9). Although the role of chromatin remodelers in transcriptional activation is well known [26–28], our results point to a role in repression under non-inducing conditions as well, although the mechanism of this repression is unknown. As the majority of genes that we designated as Taf14 or Ino80 repressed are mostly stress related genes, we can only infer that perhaps Taf14 or Ino80 repression is significant in terms of a derepression mechanism for such promoters upon transfer to inducing conditions.

**Table 7:** Significance values relating genomic properties to Taf14 regulated genes^a^

		Negatively regulated by Taf14		Positively regulated by Taf14	
Row	Mutation (Complex)	10th UP	10th DOWN	10th UP	10th DOWN
Gene specific activators					
1	*msn4* overexpression	78	…	…	…
2	*msn2* overexpression	72	…	…	…
3	*yap1* overexpression	8	…	…	…
Chromatin regulators					
4	*rpd3*∆	17	…	…	21
5	*hda1*∆	13	…	…	…
6	*tup1*∆ (Cyc8-Tup1)	40	…	…	…
7	*ino80*∆ (INO80)	100	…	…	20
8	*swi2*Δ (SWI/SNF)	39	…	…	6
9	*rsc30*∆ (RSC)	…	…	…	…
10	*gcn5*Δ (SAGA)	6	…	…	6
TBP regulators					
11	*bur6-1* mutant (NC2)	83	…	…	…
12	*TBP* ^F182V^ mutant (NC2)	26	…	…	…
13	*mot1*∆	38	…	…	…
RNA polymerase II holoenzyme					
14	*srb10*Δ (Mediator)	90	…	…	…
15	*gal11*Δ*med3*Δ (Mediator)	56	…	…	…

^a^Detailed information with calculations and percentages are provided in Additional file 2: Sheet “Taf14”

Histone amino termini can be modified post-translationally by acetylation, phosphorylation, methylation and crotonylation. These modifications affect histone charge and function. Histone acetylation is one of the best described modifications and it is mainly associated with gene activity. One such example is the acetylation of H3 and H4 tails that is usually associated with transcriptional activation [29–31]. Histone deacetylases (HDACs) Hda1 and Rpd3 are responsible for removal of acetyl tails from histone amino termini and individual deletions of these two enzymes lead to increase in histone acetylation of H3 and H4 and de-repression of a large number of genes [30]. A recent study showed that histone acetyl transferases (HATs) and HDACs regulate another dynamic mark of chromatin, histone crotonylation. These authors demonstrated that deletions of the major yeast HATs or HDACs caused alterations in the H3K9cr levels that were even more substantial than alterations in the corresponding H3K9ac levels [18]. We compared Taf14-impacted genes with the transcriptome profiles of both *hda1* and *rpd3* deacetylase mutants. Genes that show the highest levels of derepression in the histone deacetylase mutants were significantly represented among genes negatively regulated by Taf14 (Table 7, rows 4 and 5). However, we did not find a significant overlap of Taf14-repressed genes with genes that were hyper-acetylated at H3 and H4 in the *hda1*Δ and *rpd3*Δ strains (data not shown). This distinction between Hda1-mediated deacetylation and Hda1-mediated repression was previously described wherein it was found that deletion of *HDA1* always led to hyperacetylation but did not always lead to increased transcription of a particular gene [32]. It remains to be determined if overlap of Taf14-repression with genes induced in *hda1*Δ and *rpd3*Δ strains correlates with increased crotonylation of histone H3 in these strains since a recent study described the YEATS domain as currently the sole known reader of crotonyllysine [18].

The Cyc8-Tup1 repressor complex is mainly affiliated with transcriptional repression and it was shown to interact with deacetylated histone H3 and H4 tails [30, 32]. A recent study by Wong and Struhl showed that Cyc8–Tup1 complex is a general co-regulator of a class of stress-regulated, DNA binding, repressor-activator proteins that are constitutively bound to their target sites. These authors showed that Cyc8–Tup1 complex does not act as a classical co-repressor, in fact it regulates transcription primarily by masking and inhibiting the transcriptional activation domains [33]. We therefore tested whether there is an overlap of Taf14-repressed genes with those negatively regulated by Cyc8-Tup1 complex. Genes displaying the highest level of repression by Tup1 were significantly represented among Taf14-repressed genes, compared to the genome-wide average (Table 7, row 6). This correlation, together with the overlap of *taf14*Δ, *ino80*Δ and *swi2*Δ transcription profiles is interesting as a previous study showed that Cyc8-Tup1 blocks recruitment of nucleosome remodelers and therefore affects nucleosome composition of the promoter [33]. It has also been shown that Tup1 repression blocks recruitment of the Gal11 subunit of the Mediator coactivator complex. Interestingly, in addition to loss of the Tup1 repressor, proper Gal11 recruitment to target promoters also depends on histone H3 acetylation and eviction [33]. When we analysed the previously published transcriptome profile of a *gal11*Δ*med3*Δ strain, we found a very high overlap of genes induced in this mutant with a group of Taf14 and Ino80 repressed genes but no overlap with Swi2 repressed genes (Table 8). A previous study showed a similar functional interaction between Gal11 and Taf14 wherein suppression of a *gal4* deletion mutant by high levels of Gal11 required an active Taf14 protein [13]. Overlapping Tup1, Gal11 and Taf14 repression profiles as evidenced in this analysis suggest a strong link in the mechanism of repression by these three factors on a distinct set of stress responsive genes.

**Table 8:** Significance of overlap for the sets of genes repressed by each regulator pair^a^

	Taf14	Srb10	Ino80	Bur6	Tup1	Mot1	Hda1	Rpd3	Swi2
Srb10	90								
Ino80	100	88							
Bur6	83	55	80						
Tup1	40	75	45	48					
Mot1	38	26	24	83	16				
Hda1	13	26	16	15	144	7			
Rpd3	17	34	47	42	22	41	20		
Swi2	39	62	28	23	39	13	8	25	
Mediator tail	56	43	17	38	27	31	13	…	…

^a^Detailed information with calculations and percentages are provided in Additional file 2

NC2 and Mot1 are two TBP regulators that have been implicated in the stress response and target many of the same genes [34, 35]. NC2 consists of two subunits, Bur6 and Ncb2 and inhibits the recruitment of TFIIA and TFIIB to the promoter, most probably by binding to a TBP/promoter complex [36, 37]. Mot1 repression also targets TBP, in particular it dissociates TBP from DNA by using the energy of ATP hydrolysis to dissociate TBP from DNA [38, 39]. To address whether these two regulators have an overlapping regulatory role with Taf14 we looked at those genes that were most impacted by *bur6-1* and *mot1∆* mutations. As indicated in Table 7, rows 11–13, a set of genes that increased the most in *bur6-1* and *mot1*∆ mutant strains displayed a strong overlap with genes induced in a *taf14*∆ mutant. In comparison to 6% of genes that are designated as negatively regulated by Bur6 in the genome as a whole, among Taf14-repressed genes we find that 37% of these genes are also Bur6-repressed (Additional file 2: Sheet “Taf14”). We also find a strong correlation between Bur6 and Mot1 repressed genes with genes repressed by Ino80, Tup1 and Mediator tail (Gal11-Med3) (Table 8). These results are consistent with a previous study that connects NC2 regulation with the Mediator tail module wherein it was shown that proper de-repression/activation of NC2 regulated genes was dependent on the presence of Med2, Med3 and Gal11 Mediator subunits [40].

Srb10 is a kinase component of the Mediator complex and many previous studies connect this factor to repression of different elements engaged in promoting a stress response. Firstly, Srb10 phosphorylates the C-terminal domain of pol II which leads to transcription repression [41]. Secondly, Srb10 inactivates several transcription activators that promote a stress response. In this case phosphorylation leads to elimination of these transcriptional activators from the nucleus [42, 43]. Thirdly, several studies connected Srb10 to repression by Cyc8-Tup1 repressor complex [32, 44]. Based on these previous studies and our results in this study linking Taf14 and Tup1 repression we next examined whether Taf14-repressed genes show overlap with genes impacted by Srb10 dysfunction. Genes that are significantly repressed by Srb10 showed a very high overlap with genes repressed by Taf14 (Table 7, row 14). Moreover, we found that 44% of Taf14-repressed genes were also induced in a *srb10*Δ mutant strain whereas across the whole genome only 6% are Srb10-repressed. Thus, we propose that Srb10 as an important factor in the regulation of Taf14-repressed genes (Additional file 2: Sheet “Taf14”). Msn2 is one of the stress activators targeted by Srb10 repression. Therefore, we also examined genes regulated by the stress activators Msn2 and Msn4 and found a high degree of overlap between Taf14-repressed genes and a set of genes positively regulated by Msn2/4 (Table 7, rows 1–3).

The combined results of these analyses support the hypothesis that Taf14 is an important negative regulator in a coordinated stress response pathway (Table 8, Additional file 1: Table S3) that is upregulated by the coactivator complex SAGA and stress activators such as Msn2/4 and downregulated by Srb10, histone deacetylases and Tup1-dependent repression mechanisms.

### Taf14 co-occupies promoters with a distinct set of factors from Cyc8-Tup1-regulatory network

Previous studies have described Taf14 as a protein that physically associations with a number of multi-subunit transcriptional regulators including the general transcription factors TFIID and TFIIF, the chromatin-modifying complexes SWI/SNF, INO80, RSC and NuA3 and the coactivator complex Mediator [9–11]. We next analysed previously published whole genome occupancy data for 200 transcription-related proteins [21] in order to correlate Taf14 co-occupancy at the genome-wide level. In Table 9 we list the factors that show the highest statistical significance of overlap for the set of genes co-occupied by each regulator and Taf14 (logP > 100, further referred to as the high overlap group). These factors show significant occupancy overlap with Taf14 under both non-inducing and heat shock conditions. When we compare the binding locations with highest occupancy on each promoter we find that in addition to binding a similar set of promoters, these factors from the high overlap group tend to bind in close vicinity (<40 bp distant) to the Taf14 binding location (Additional file 4: Figure S1). The majority of these proteins have not been previously connected to Taf14 either physically or functionally with the exception of Taf4, a subunit of TFIID, Rvb1, a subunit of INO80 complex and Rsc8, a subunit of the RSC complex. Nevertheless, it is significant that these regulators are bound to similar promoters in the vicinity of Taf14. Interestingly, four proteins from this high overlap group are associated directly or indirectly to transcriptional regulation via the Cyc8-Tup1 complex. Three proteins, Cti6, Rxt2 and Ash1 are components of the Rpd3L histone deactylase complex which is significant as Cyc8-Tup1 interacts with deacetylated histone H3 and H4 tails [30, 32]. Cti6 was also shown to relieve transcriptional repression by binding the Cyc8-Tup1 complex and recruiting SAGA to the repressed promoter [45]. The fourth transcription factor, Skn7, physically interacts with the Cyc8-Tup1 complex and recruits Tup1 to its targets [46]. Skn7 is a nuclear response regulator required for optimal induction of heat shock genes in response to oxidative stress. The correlation of Taf14- and Tup1-repressed gene sets (Table 8) and co-occupancy data (Table 5 and 9) clearly suggest a functional relationship between Taf14 and the Cyc8-Tup1 regulatory pathways.

**Table 9:** Significance values relating whole genome occupancy to set of genes occupied by Taf14^a^

Function	Complex	Protein^b^	25UTmax	37UTmax
Chromatin remodeling	INO80,SWR1	Rvb1	151	154
	SWR1	Swc5	176	143
	RSC	Rsc8	223	231
Cyc8-Tup1 interacting	RPD3	Ash1	205	139
		Rxt2	172	184
		Cti6	167	145
	Stress related TF	Skn7	107	128
General transcription factors	TFIID	Taf4	109	130
	TFIIH	Tfb3	154	165
		Tfb1	108	156
Transcription elongation	Histone demetylase	Rph1	209	210
	THO/TREX	Hpr1	113	132
RNA polymerase III transcription	TFIIIC	Tfc6	104	106
Pre-mRNA 3’ end processing and transcription termination	CF IA	Pcf11	102	112
Activation of ribosomal genes transcription	Pol II TF	Sfp1	146	146
Histone H3 metylation	COMPASS	Bre2	106	113

^a^Detailed information with calculations and percentages are provided in Additional file 3: Sheets “25UTmax” and “37UTmax”

^b^Only proteins that show the highest statistical significance of overlap for the set of genes co-occupied by each regulator and Taf14 are displayed, that is those with logP values greater than 100, under both non-inducing and heat shock conditions

Two proteins from the Taf14 high overlap group were shown previously to regulate transcription elongation; Rph1, is a histone demethylase that associates with actively transcribed regions [47] and Hpr1, a subunit of the THO/TREX elongation complexes [48, 49]. The co-occupancy of Taf14 with these factors may be indicative of a complex role in transcription elongation as described in earlier studies [12, 50–54].

## Discussion

Taf14 is a member of the YEATS domain superfamily and a subunit of multiple chromatin-modifying and transcription complexes [10]. Genome-wide analysis of *taf14*Δ strains shows that distinct sets of genes can be either positively or negatively regulated by Taf14 [15, 20], however the mechanistic aspects of this regulation are unknown. In this report we focused our analysis on genes that are negatively regulated by Taf14 as this aspect of its function has not been reported on before.

We analysed the genome-wide impact of *taf14* deletion and our results clearly show that Taf14-repressed genes tend to be extensively regulated, in a positive manner by SAGA complex and stress-responsive activator proteins, and negatively by a number of repressors that act upon promoter chromatin and TBP. The correlation of Taf14-dependent genes with those regulated by SAGA is further corroborated by the recent report of synthetic lethality when *taf14* deletion is combined with a *gcn5* mutation [15]. Interestingly, even though Taf14 is a subunit of TFIID, we did not find any correlations of Taf14-dependent and TFIID-dependent genes under normal conditions. This is perhaps expected as Taf14 is non-essential under normal conditions while the remaining Tafs are essential genes. However, it remains possible that Taf14 has an essential function within TFIID under some other condition which has yet to be tested.

A *taf14* deletion strain is sensitive to multiple stressors including heat stress. Our analysis of genome-wide occupancy of Taf14 under non-inducing and heat-stress conditions showed that on a global level, under non-inducing conditions, Taf14 is found predominantly at transcription start sites and upon heat stress a global relocalization occurs to predominantly UAS sites. This suggests that in addition to a repressive role under non-inducing conditions, Taf14 may be important for activation under stress conditions through its localization at UAS sequences.

**Fig. 1:**
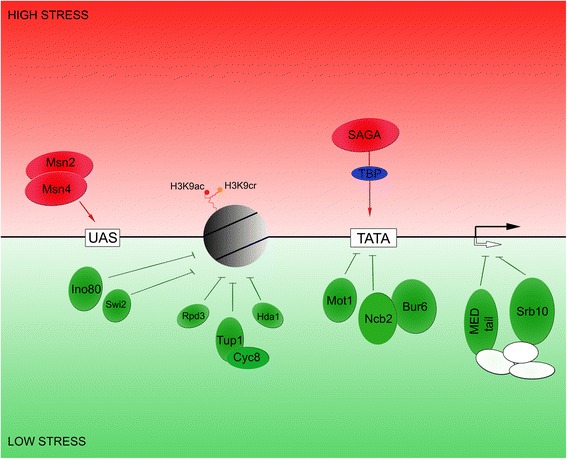
Taf14 negatively regulated genes show distinct regulatory features. These genes are mostly stress-related, their promoters mainly contain a TATA box and SAGA complex is mostly responsible for loading TBP to these promoters. Transcription of these genes is also highly regulated by a distinct set of regulators. Besides positive regulation by the stress related activator Msn2/4, transcription regulation of Taf14 repressed promoters is dominated by negative regulators of transcription. These include the negative co-regulators histone de-acetylases (Hda1 and Rpd3), TBP repressors Mot1 and NC2 (Ncb2-Bur6), Cyc8-Tup1 repressor complex, and two members of Mediator complex, Srb10 and the Mediator tail module. Repressing elements of transcription are illustrated in red and activating elements are in green

Given the significant presence of Taf14-repressed genes among those induced during various types of stress, and also the very strong overlap with negative regulation by Srb10, Cyc8-Tup1, NC2, Mot1 and Mediator tail module (Fig. 1), we propose that Taf14, either through direct or indirect interaction with these factors keeps a distinct set of genes repressed under non-inducing conditions. Surprisingly, with the exception of Mediator, none of the aforementioned negative regulators has previously been implicated to interact with Taf14 either physically or functionally. Notably, we found that *taf14* deletion led to de-repression of genes required for a variety of stress responses and yet the *taf14* deletion strain remains sensitive to these stresses. This suggests that de-repression by simply removing Taf14 is not sufficient to allow the deletion strain to grow in the presence of stressors and that the presence of Taf14 may be required further for proper activation of stress responsive genes. We suggest that Taf14-dependent repression is important for proper de-repression/activation under inducing conditions as reported above for the case of MMS induction of DNA-damage response genes (Table 2, row 9). We also analyzed genome-wide ChIP data for occupancy of known transcription factors upon shift from normal to acute heat-shock conditions. Our analysis of three different datasets shows that the described overlap of transcription profiles from distinct transcription factor deletion strains (Table 7 and 8) is strongly accompanied by an overlap of their genome-wide occupancy (Table 3–5). Firstly, promoters of Taf14-repressed genes strongly overlap with promoters that showed the largest increase in occupancy of INO80, NC2 and Cyc8/Tup1 upon heat shock (Table 3). Secondly, promoters that showed the largest increase of Taf14 occupancy upon heat shock are mostly promoters of genes that by many elements can be attributed to the SAGA-regulatory pathway (Table 4). Thirdly, upon heat shock Taf14 tends to show increased binding to a similar set of promoters as do Cyc8-Tup1, Mediator and INO80 (Table 5). In our survey of proteins that co-occupy a similar set of promoters as Taf14 across the genome, we found a group of proteins that not only bind a highly similar set of promoters as Taf14 but also tend to bind in close vicinity to the Taf14 binding location (Table 9 and Additional file 4: Figure S1). Among these proteins at least three complexes, TFIID, INO80 and RSC, are known to have Taf14 as a subunit. In addition four proteins, Skn7, Cti6, Rxt2 and Ash1 from the high overlap group belong to a group of factors that directly or indirectly regulate transcription via the Cyc8-Tup1 repressor complex which is consistent with our transcriptomic analysis showing co-regulation of Taf14- and Cyc8-Tup1-repressed genes. Thus, our combined data suggests a new and previously unrecognized interaction of Taf14-dependent gene regulation and regulation via Cyc8-Tup1. It remains to be examined whether this interaction is mediated by known complexes containing Taf14 or whether Taf14 acts alone or as part of a complex not previously associated with Taf14.

A recent study has shown that, among acetylated histone marks, the Taf14 YEATS domain preferentially binds the H3K9Ac histone residue with no significant binding of unacetylated histones [15]. However, more recently the Taf14 YEATS domain has been identified as the first reader of histone H3 crotonyllysine (H3K9cr) and it has been shown that H3K9cr is preferred over H3K9Ac. These authors further demonstrated that H3K9cr exists in yeast and is dynamically regulated by HATs and HDACs [18]. In this study we have shown an overlap of Taf14-repressed genes with those genes that show the highest levels of derepression in the histone deacetylase mutants (*hda1* and *rpd3*). Thus Rpd3 and Hda1 are additional negative regulators of chromatin that correlate with transcriptional repression by Taf14. Taf14-repressed genes do not appear to correlate with hyperacetylation in the *hda1* and *rpd3* dependent genes although it may be that a correlation with crotonylation remains to be examined.

Previous reports have implicated the human YEATS domain homolog AF9 in regulation of transcription elongation [52–54] and thus it is interesting that two yeast proteins in the Taf14 co-occupancy high overlap group, Rph1 and Hpr1, are transcription elongation regulators. A connection between Taf14 and regulation of transcription elongation was noted in previous studies, one showing that the absence of Taf14 in the cell appears to create a dependence on an undefined function of eukaryotic transcription elongation factor TFIIS, mediated by its N-terminal region [12] and two more recent studies looking at a TFIIF and TFIIS connection in promoting transcript elongation by RNA polymerase II [50, 51]. A question that remains is whether Taf14 is involved in regulating dynamics of transcriptional elongation in yeast, similarly to AF9 and ENL [52–54], by recruiting different elongation-promoting factors to the RNA pol II elongation complex.

Our genome-wide study describes a highly networked regulatory role of the YEATS domain protein Taf14. Considering the recently identified interactions of Taf14’s YEATS domain with acetylated and preferentially with crotonylated histone H3 [15, 18], an emerging model of Taf14 action suggests it acts as a regulatory bridge between chromatin and the complexes that act upon it and that it may have a role in integration of signaling networks. The C-terminal of Taf14 contains a conserved ET-domain also found in AF9 (known as the ANC1 homology domain (AHD)) [55, 56]. The AHD domain is intrinsically disordered and has been shown to facilitate the dynamic exchange of high affinity interaction partners. The differing functions of the Taf14 binding partners [10] suggest that the role of its intrinsically disordered ET domain, similarly to the AHD in the homologue AF9 [56], may be to promote exchange of these Taf14 interacting factors subsequent to changes in their local concentration and/or affinity. Given that the known histone modifications recognized by the Taf14 YEATS domain have not been associated with transcriptional repression it is unclear whether this type of mechanism also applies to Taf14-dependent gene repression.

## Conclusion

Our analyses have shown that Taf14 is an important negative regulator of stress-responsive gene transcription. Our data and the work of others suggest that Taf14 also plays a role in transcriptional activation of the stress response as well. Taken together our analyses suggest a novel role for Taf14 in repression and derepression of stress induced genes, most probably as part of a regulatory network which includes Cyc8-Tup1, Srb10 and histone modifying enzymes. This genome-wide survey of transcriptome and ChIP data has uncovered novel aspects of co-regulation of Taf14-repressed genes and should inform future experimental studies that will mechanistically define Taf14-dependent repression in regulating yeast gene transcription.

## Additional files

Additional file 1: Tables S1-S5. Supplementary meta-analysis tables, data sources for meta-analyses. (XLSX 103 kb)
Additional file 2: List of genes and calculations of transcription data. (XLS 227 kb)
Additional file 3: List of genes and calculations of occupancy data and cross-study of occupancy and transcription data, location linkage analysis for Figure S1. (XLSX 280 kb)
Additional file 4: Figure S1. Location linkage of Taf14 and proteins co-occupying high number of promotors (-logP > 100). (PDF 154 kb)

## Abbreviations

ChIP: Chromatin immunoprecipitation
GTF: General transcription factor
HAT: Histone acetyl transferase
HDAC: Histone deacetylase
MMS: Methyl methanesulfonate
PIC: Pre-initiation complex
TSS: Transcription start site
UAS: Upstream activating sequence
