# Supplementary material for: A meta-analysis reveals complex regulatory properties at Taf14-repressed genes
Source: BMC Genomics. 2017 Feb 16;18:175. doi: 10.1186/s12864-017-3544-6 (PMC5312515; doi:10.1186/s12864-017-3544-6)
Supplement: Additional file 4: Figure S1. — Location linkage of Taf14 and proteins co-occupying high number of promotors (-logP > 100). (PDF 154 kb) [file 12864_2017_3544_MOESM4_ESM.pdf]

Figure S1 Location linkage of Taf14 and proteins co-occupying high number of promotors (-logP>100)[a]

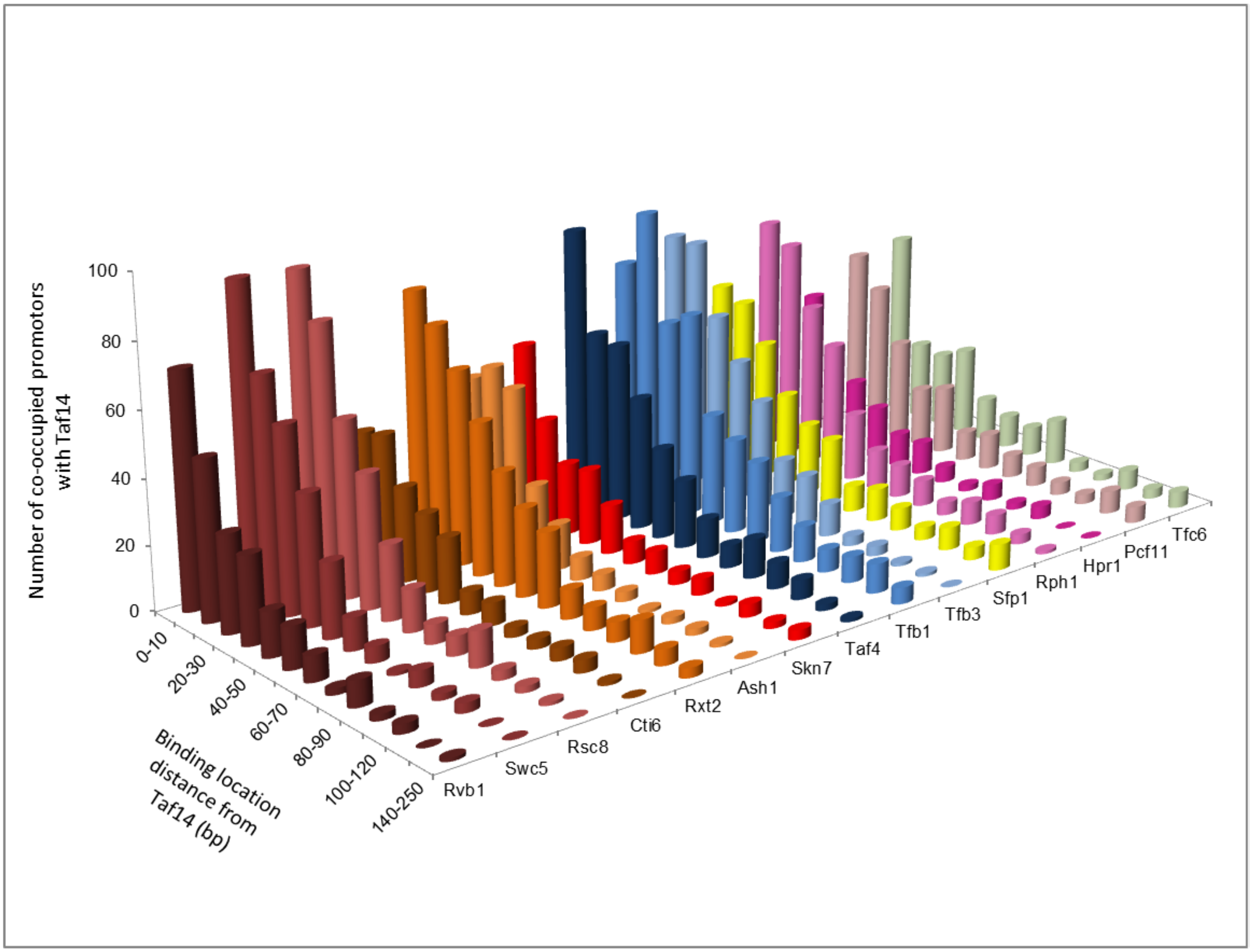

[a]Detailed information with calculations and percentages are provided in Additional file 3: Sheet “Location linkage”.
